# Advances in Wearable Biosensors for Healthcare: Current Trends, Applications, and Future Perspectives

**DOI:** 10.3390/bios14110560

**Published:** 2024-11-18

**Authors:** Dang-Khoa Vo, Kieu The Loan Trinh

**Affiliations:** 1 College of Pharmacy, Gachon University, 191 Hambakmoe-ro, Yeonsu-gu, Incheon 21936, Republic of Korea; vodangkhoa135@gmail.com; 2 BioNano Applications Research Center, Gachon University, 1342 Seongnam-daero, Sujeong-gu, Seongnam-si 13120, Republic of Korea

**Keywords:** wearable biosensor, bioanalysis, telemedicine, personalized health management, lifestyle

## Abstract

Wearable biosensors are a fast-evolving topic at the intersection of healthcare, technology, and personalized medicine. These sensors, which are frequently integrated into clothes and accessories or directly applied to the skin, provide continuous, real-time monitoring of physiological and biochemical parameters such as heart rate, glucose levels, and hydration status. Recent breakthroughs in downsizing, materials science, and wireless communication have greatly improved the functionality, comfort, and accessibility of wearable biosensors. This review examines the present status of wearable biosensor technology, with an emphasis on advances in sensor design, fabrication techniques, and data analysis algorithms. We analyze diverse applications in clinical diagnostics, chronic illness management, and fitness tracking, emphasizing their capacity to transform health monitoring and facilitate early disease diagnosis. Additionally, this review seeks to shed light on the future of wearable biosensors in healthcare and wellness by summarizing existing trends and new advancements.

## 1. Introduction

Wearable biosensors are a class of non-invasive devices embedded in smartwatches and patches, among other things, designed to monitor vital physiological and biochemical signals in real time [1]. This would be achieved through continuous monitoring of heart rate, glucose level, body temperature, and more while offering the user instant feedback and enabling long-term accumulation for personalized health management [2]. They are an essential part of chronic disease management, such as diabetes and cardiovascular diseases [3]. This, in turn, has created an exponentially higher demand for wearable biosensors that can monitor the patient continuously and remotely to minimize doctor visits. Recent developments in flexible biocompatible materials have made them more comfortable to wear, thus enabling such extended wear [4,5]. These devices also generate voluminous health data. Linked to artificial intelligence (AI) and machine learning, this allows for predictive healthcare examples, identifying potential risks well before symptoms manifest [6]. In the realm of health and wellness, there has come about the use of wearable biosensors for tracking activity, sleep, and other general health metrics. Overall, these devices support much more personalized and preventive healthcare, which should reduce costs while improving outcomes by empowering individuals to take a more proactive approach toward their health.

This paper provides an extensive review of wearable biosensors regarding recent developments, current trends, key technologies, and novel applications in health, fitness, and wellness. This paper aims to demonstrate how the new development in sensor material, design, and integration with AI could mark a sea change in personalized health monitoring and chronic disease management. Concomitantly, the paper also explores the increasing trend in the use of wearable biosensors in telemedicine and remote patient care in the context of pandemic and post-pandemic health care. In addition to reviewing present applications, the paper then goes on to describe perspectives for the future, emerging technologies, and possible applications. The paper also underlines how wearable biosensors might become one of the major forces in shaping the future of healthcare toward more preventive, data-driven, and patient-centered solutions.

## 2. Overview of Wearable Biosensors

### 2.1. Types of Wearable Biosensors and Key Technologies

Various wearable biosensors are available. Each of them was created for specific applications in a manner that they measure specific biological or biochemical functions and then transform the message into electronic output that can be measured. Among these types are electrochemical biosensors, optical biosensors, and piezoelectric biosensors [7,8,9]. Firstly, electrochemical biosensors are devices that are used in the detection of biochemical substances such as glucose or lactate using electrochemical reactions, converting the concentration of the target molecule into electrical impulses. They are found in appliances such as glucose meters. Secondly, optical biosensors are devices that utilize light to find out the presence of biomolecules by detecting changes in light absorption, fluorescence, or scattering. Typically, they are a component of a pulse oximeter used in the measurement of blood oxygen levels. Thirdly, piezoelectric biosensors are gadgets that are based on the piezoelectric effect that show the changes in mass, pressure, or mechanical stress and convert them into electrical signals. They are widely used in tracking physical activities and breathing. Different biosensors will contribute to real-time, non-invasive health monitoring, which is essential in different healthcare, fitness, and wellness applications. Every one of these types of biosensors is designed in the case of specific applications. That is why the advantages of biosensor technology for health purposes are diverse and significant as well.

The functional aspect of wearable biosensors is facilitated by different key technologies that lead to emphasis in terms of their accuracy, flexibility, and integration into wearable devices. The first key technology to consider is microfluidics. The technological breakthrough in this aspect is the ability to use a chip to control and manipulate minuscule amounts of fluids [10]. Microfluidics is a technology that allows one to securely obtain fluids such as sweat, saliva, or interstitial fluid using non-invasive methods. It provides uninterrupted real-time glucose measurement and continuous monitoring of the level of electrolytes and lactate. A second important key technology is flexible electronics. Regarding comfort and continuous use, wearable biosensors manage to achieve this thanks to their flexible and stretchable electronics. These materials are really thin and lightweight, and thus, they properly fit into the skin and can also be twisted or stretched without the loss of functionality; so indeed, they are ideal for carrying out health monitoring in real time [11]. Furthermore, the use of nanomaterials such as graphene and carbon nanotubes largely contributes to the sensitivity and selectivity of biosensors [12]. The use of these nanomaterials ensures that the biological markers are identified more precisely in the lower concentration, and thus it may improve the measurement accuracy. Moreover, tools such as Bluetooth, Near Field Communication (NFC), and Wi-Fi make it possible for wireless communication between biosensors and mobile devices, and, therefore, real-time monitoring of health and remote data access is allowed [13]. Lastly, attention must be given to the technology of energy harvesting. The technology powers itself off action taken by the user and gathers power from body movements for permutations of the system based on body heat and light intensity in the living area. Such inventions have become quite important in the attempt to improve battery life and decrease the frequency of charging to almost zero [14]. These enabling technologies are critical for the development of wearable biosensors that are reliable, comfortable, and efficient for real-time health monitoring.

### 2.2. Materials and Design of Wearable Biosensors

The performance and effectiveness of wearable biosensors are mostly dependent on the materials used to manufacture them [15]. Among the materials that are commonly used in this technology are polymers, nanomaterials, and textiles, as well as electronic inks and hydrogels. A polymer that is biocompatible and mainly made of polyethylene and polydimethylsiloxane (PDMS) is usually the material of choice due to its flexibility, strength, and ease of manufacture. The polymer materials can be modified to form thin films or microstructures that are necessary for sensor functionality, which makes them suitable for skin contact applications [5,16]. Materials that are nanosized, such as carbon nanotubes, graphene, and metal nanoparticles, can greatly enhance the sensitivity and specificity of biosensors. The nanoparticles can have a very large surface area, and thus the interactions with the biomolecules are very effective, leading to highly sensitive sensors, even at low concentrations [17]. Smart textiles make up the conductive fibers or materials in the fabric that produce the wearable biosensors that are difficult to see and very comfortable to use. Hence, the textiles can be expanded to a higher physiological level, such as heart rate or muscle activity, while still being lightweight and flexible [18,19]. Conductive inks made of silver or carbon that are used in electronic fabrication are those that can be used on flexible substrates to make lightweight sensors with properties such as adaptability. This allows for the mass production of biosensors at a low price, which can be any shape or size [20]. Hydrogel is one of the water-based polymers mostly used, as it can be used in harmony with biology, and it can be like real body tissue. In this paper, hydrogel refers to sensor construction; it becomes the mediator between the sensor and the target analytes, which makes it useful in certain applications such as sweat analysis [21]. The combination of these materials is what gives us the wearable devices, mostly biosensors that monitor signals in real-time, being comfortable and the most user-friendly to the person that wears it.

**Figure 1:**
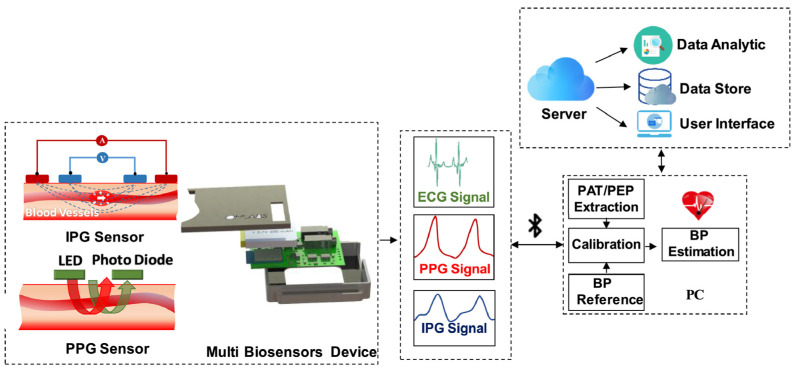
Overview of the multimodal body sensors for BP estimation with IoT application. Copyright Elsevier (2024) [28].

The wearable biosensors are designed to be very thin, flexible, and small by using technologies such as, for instance, stretching, folding, and miniaturization. Bendy and stretchy biosensors are specially designed to fit the body and its natural movements perfectly, so they do not cause any inconvenience. With their flexible and stretchable designs, they act as a kind of conductor, and their manner of use can be likened to that of an elastomer, which wearers can easily use with sensors on the skin without detaching them [22]. These sensors can change, and the adaptation of the skin is guided by electricity and magnetism through the conductive polymers and elastomer fiber materials [23,24]. Miniaturization is the other essential feature of making wearable biosensors compact and available to everyone [22]. Advances in microfabrication techniques have allowed for the development of sensors of significantly smaller sizes without losing their effectiveness. By decreasing the size of individual sensors in a wearable device, and thus the total weight and bulkiness, users can easily carry them, saving their bodies and maintaining healthy conditions [25]. The size of the moving part also allows for the integration of various sensors into a single device, which in turn enables the concurrent collection of different aspects of the physiological parameters. The seamless pairing of biosensors with smart wearable devices, such as smartwatches, wristbands, and intelligent clothing, for example, enables the sports-conscious person to have a close relationship with these gadgets as they possess a high level of accuracy and user-friendliness [26]. Successful collaborations with these devices involve quick and accurate recording, as the majority of gadgets deliver real-time information to customers via interactive mobile applications [27]. Additionally, the use of wireless communication technologies, such as Bluetooth and NFC, ensures that data can be transmitted seamlessly to smartphones or cloud platforms for storage and further analysis (Figure 1) [28]. This joint work between biosensors and wearable technologies encourages people not only through long user interactions but also through health monitoring. Altogether, the commitment to the creation of wearable biosensors that feature flexible, stretchable design, miniaturization, and easy integration into the wearable device is fundamental to the betterment of the wearability and functionality of wearable biosensors in the user’s everyday life.

Power supply and data transmission become necessary if the functionality and practical usage of wearable biosensors are taken into consideration. Wearable biosensors have the fundamental challenge of supplying appropriate, energy-efficient power sources so that the device can operate continuously without the need for frequent refueling [14,29]. Compact lithium-ion batteries are used in many devices; however, there is increasing utilization of developments in energy-harvesting technologies [29,30]. These methods can exploit body heat, motion, or ambient light for the derivation of energy and thus allow sensors to operate autonomously over a longer period than previously experienced [2]. In addition to this, optimized energy use can be achieved through low-power electronics and sleep mode systems, highly improving battery life and thus making wearables more comfortable for users [31]. To ensure continuous, uninterrupted data transfer, wearable biosensors mostly incorporate wireless means of communication. Examples are Bluetooth, NFC, Wi-Fi, LoRa, and Zigbee. Bluetooth works perfectly for transceivers, as it has a low power rating and high reliability [32]. This enables the management of communication between wearable biosensors and smartphones as well as other devices. It is frequently used in fitness trackers and smartwatches that allow the analysis and synchronizing of user data in real time [33]. NFC is a contactless communication that allows two devices near each other to peer naturally. This is useful for applications involving the transfer of data within short distances and within nanoseconds, such as in payment systems or device pairing [34]. In this case, data may be transferred from a wearable device to another device simply by tapping the two together without the use of any buttons [35]. Wi-Fi can be deployed in cases when data are required to be transmitted faster. Even though it requires more energy compared to Bluetooth, its faster speed of transmission comes in handy in cases where large volumes of data need to be accessed instantly [36]. Moreover, LoRa and Zigbee are known as low-power, wide-area network technologies generally used in low-power, long-range communication for IoT applications [37]. They enable communication effectively while conserving a lot of energy. This is an added advantage for biosensors that are incorporated in health monitoring systems for patients that are not within reach [38]. The use of appropriate power management techniques coupled with strong wireless communication technologies allows the day-to-day development of wearable biosensors that can afford a user continuous health monitoring while allowing instant access to health information for the user, which improves the user’s experience and encourages health management behaviors.

## 3. Current Trends in Wearable Biosensors

### 3.1. Real-Time Health Monitoring and Applications in Monitoring Vital Signs

One of the most significant benefits is probably how real-time health monitoring has become mostly facilitated by wearable biosensors for checking important vital signs and thereby managing fitness and health. Such devices offer real-time, non-invasive outputs that allow users to visualize their physiological status and empower them to make informed healthcare decisions. The applications in the monitoring include glucose monitoring, heart rate monitoring, pulse rate monitoring, oxygen saturation monitoring, blood pressure monitoring, body temperature monitoring, and activity and sleep monitoring.

For glucose monitoring, biosensors worn on the body, such as continuous glucose monitors (CGMs), give real-time blood sugar readings and can be a boon for anyone with diabetes [39,40,41]. These devices measure the glucose concentration in interstitial fluid and allow users to better manage their insulin intake or menu choices, which reduces the risk of hypoglycemia or hyperglycemia. Furthermore, for heart rate monitoring, modern typical fitness trackers and smartwatches can track the user’s heart rate by utilizing photoplethysmography (PPG) or electrocardiography (ECG) to provide feedback. They offer an overview of heart rate variability in real time to enable users to track fluctuations in their cardiovascular health, better tailor physical activity, and identify possible arrhythmias or other heart- and circulatory-related anomalies [42,43]. In oxygen saturation monitoring, pulse oximeters are devices you wear that check your blood oxygen levels (SpO_2_) using light absorption methods [44]. People with breathing problems, such as chronic obstructive pulmonary disease (COPD) or asthma, must always keep an eye on their oxygen levels. This helps doctors to step in when needed and tweak treatment plans. In regard to blood pressure monitoring, new wearable technology now includes ways to check blood pressure, giving users a look at their heart health without needing the old cuff-style devices [45]. Tracking blood pressure continuously can spot high blood pressure and encourage people to see their doctor sooner [46]. Moreover, wearable biosensors can also monitor body temperature as it occurs, which is useful for checking fevers or other health issues [47]. Ongoing temperature information can help manage sicknesses and show patterns in overall health. Although they are not vital signs, many wearable biosensors keep records on how much you move and sleep, giving useful information about your overall health [48,49,50]. Knowing about your activity and sleep quality can help you tweak your lifestyle to make your body work better. Using wearable biosensors to watch your health in real time makes it much easier to take care of yourself. By always showing you important health signs, such as sugar levels, heart rate, and oxygen in your blood, these gadgets help you take charge of your health. This can lead to better results and a happier life.

### 3.2. Non-Invasive Sensing, Key Advances in Non-Invasive Sensing, and Minimally Invasive Sensing

**Figure 2:**
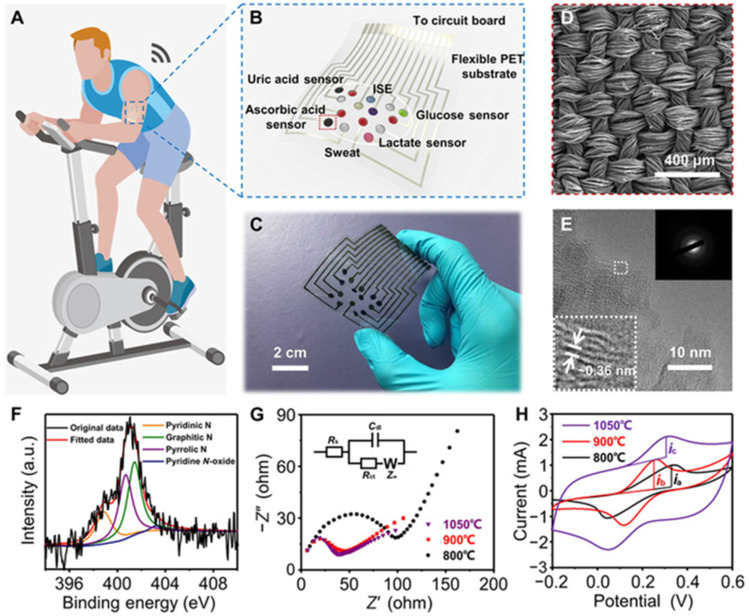
Wearable sweat analysis patch based on SilkNCT, an integrated textile sensor patch for real-time and multiplex sweat analysis. (**A**,**B**) Schematic illustration of wearable sweat analysis patch mounted on human skin (**A**) and the multiplex electrochemical sensor array integrated in the patch (**B**). (**C**) Photograph of the wearable sweat analysis patch. (**D**,**E**) SEM (**D**) and TEM (**E**) images of the carbonized silk fabric, showing its hierarchical woven macrostructure and microcrystalline graphite-like microstructure, respectively. (**F**) High-resolution XPS spectrum of N1s for the carbonized silk fabric. a.u., arbitrary units. (**G**) EIS of the carbonized silk fabric prepared at different temperatures. Inset in (**G**) shows an equivalent circuit model. (**H**) Cyclic voltammograms of the carbonized silk fabric prepared at different temperatures in 0.1 M KCl solution containing 5.0 mM [Fe(CN)_6_]^3−/4−^. Copyright AAAS (2019) [53].

The ability to attain physiological data without conventional blood samples has revolutionized health monitoring thanks to developments in non-invasive sensor technologies. The new trend leans toward painless, non-invasive ways that measure vital signs using bodily fluids, including saliva, sweat, and interstitial fluid in the skin. First, the use of biosensors that are based on sweat and may be worn on the body allows for the monitoring of several different health indicators [51,52]. The presence of electrolytes, glucose, lactate, and other metabolites may be detected by sweat sensors, making it an excellent predictor of hydration, activity level, and metabolic status (Figure 2) [53]. Sweat is a fantastic diagnostic of these things. As a result of recent developments in electrochemical sensors and microfluidics, sweat analysis has lately become more sensitive and specific [54]. This has made it possible to perform real-time monitoring while participating in physical activity or going about daily activities. Second, saliva-based biosensors have attracted significant attention due to their capacity to identify indicators of systemic health, including glucose, hormones, and infections [55]. These sensors are capable of monitoring a wide range of variables, such as an individual’s metabolic status, oral health, and hormonal levels, through the use of methods such as optical detection and electrochemical analysis [56]. This method is particularly appealing for routine health checks and disease monitoring due to its ability to capture saliva samples without requiring any invasive procedures [57]. This renders it an optimal option for these procedures. Third, the development of flexible and stretchable electronics has enabled the creation of skin-based sensors that can monitor interstitial fluid or skin temperature [22,58]. These sensors are capable of identifying specific biomarkers that may suggest potential health issues, in addition to providing real-time data on pH and hydration levels [22,59]. They are appropriate for continuous health monitoring due to their form-fitting design, which allows for extended use. The incorporation of these non-invasive sensing technologies into wearable devices, such as fitness trackers and smartwatches, makes it easier for users to receive information regarding their health. These devices provide a comprehensive perspective of the wearer’s health status by combining numerous sensing modalities, such as monitoring the wearer’s heart rate and sweat [27,37]. Advancements in nanoscale materials and microfabrication techniques have markedly improved the accuracy and reliability of non-invasive sensors [27,60,61]. These developments enable the detection of biomarkers in saliva and sweat at low quantities, enhancing the potential for early illness identification and personalized health management.

Otherwise, minimally invasive technologies in wearable biosensors offer a middle ground between non-invasive and fully invasive methods, enabling accurate, real-time health monitoring with minimal discomfort. Major breakthroughs are represented by the microneedle patches that access interstitial fluid for glucose and electrolyte monitoring, and, on the other hand, the flexible epidermal electronics and hydrogel-based sensors that stick to the skin to measure hydration, pH, and other such biomarkers found in sweat [62]. Bioelectronic “smart tattoos” [63] and thin, stretchable electronics are newer approaches to enabling unobtrusive, long-term monitoring of physiological metrics and establishing a position in chronic disease management, fitness, and lifestyle tracking. While promising, these technologies face challenges in establishing long-term accuracy and overcoming regulatory approvals, so more research needs to be performed before these technologies increase in reliability and usability. Tehrani et al. [64] introduced a new wearable device using a microneedle array to achieve the real-time monitoring of several biomarkers by sampling interstitial fluid (ISF). The new technology is non-invasive and avoids blood drawing, as the microneedles only break the skin enough to access ISF, which does not cause significant pain. It incorporates biosensors in the microneedles that allow, in real time, the monitoring of very important biomarkers, such as glucose, lactate, and pH, among others, in the follow-up of diabetes, metabolic disorders, and sepsis. The sensing elements utilize electrochemical techniques, offering high sensitivity and specificity for each biomarker; further, because of the array design, simultaneous and multiplexed measurements are possible. Among the benefits, real-time data collection can seriously enhance the provision of tailored health care by giving instant insights into the metabolic and health status of a patient. The technology also supports remote monitoring and could reduce frequent hospital visits. Moreover, Yang et al. [65] produced unprecedented wearable technology that monitors cell-free DNA continuously and in a non-invasive way. It achieves high accuracy with CRISPR-Cas9 technology through its microneedle patch by selectively capturing and identifying cell-free DNA sequences in real time. More importantly, it allows for real-time continuous monitoring of genetic biomarkers associated with a variety of conditions, such as cancer. The CRISPR-Cas9 system in the patch is extremely sensitive and specific for detecting genetic mutations or changes over time with a high degree of accuracy. This development has great potential for personalized medicine, due to its offering of early detection and timely monitoring of disease progression in a convenient, minimally invasive format. The wearable platform increases not only patient comfort and compliance but also opens up new possibilities for proactive disease management and preventive healthcare.

As minimally invasive and non-invasive sensing technologies continue to develop, they represent a big step forward in the field of health monitoring. These technologies offer alternatives that are more convenient and are more pleasant compared to blood samples. Through the utilization of biological fluids such as sweat, saliva, and interstitial fluid, these advancements not only alleviate discomfort but also make it possible to monitor critical health factors in a continuous and real-time manner. This gives individuals the ability to take charge of their health and take responsibility for their well-being.

### 3.3. Artificial Intelligence (AI) Integration and Key Contributions of AI and Machine Learning

**Figure 3:**
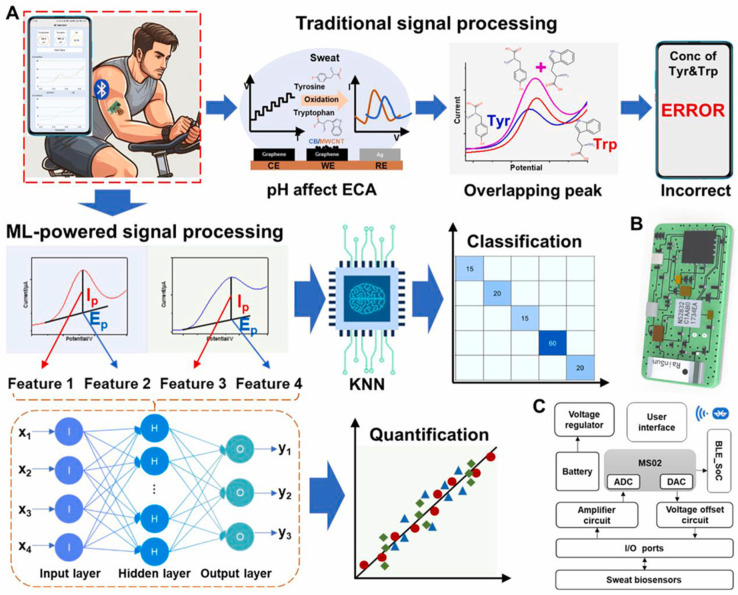
Schematics and images of the machine-learning-powered wearable sensor for distinguishable and predictable sensing. (**A**) Schematics of machine-learning-powered signal processing overcome the limitation of traditional signal processing, achieving accurate classification and quantification. CE, counter electrode; WE, working electrode; RE, reference electrode; ECA, electrochemical catalytic activity; KNN, k-nearest neighbor. (**B**) Schematic of micro-electrochemical system. (**C**) System-level block diagram showing the signal transduction, processing, and wireless transmission from the sensors to the user interface. ADC, analog-to-digital converter; DAC, digital-to-analog converter; I/O, input/output; BLE SoC, Bluetooth low energy system on chip. Copyright Elsevier (2024) [66].

The integration of AI and machine learning into wearable biosensors has added a new dimension to health monitoring: enhancing predictive accuracy and offering personalized feedback to users (Figure 3) [66]. These technologies thereby provide the biosensor with the ability to analyze large volumes of data, recognize patterns, and make informed predictions regarding an individual’s health status. The AI algorithms learn both from historical and real-time wearable biosensor data to identify trend analysis in health outcomes and predict future states [67,68]. For instance, given some heart rate variability, AI may predict possible cardiac events and thus enable timely interventions [69]. Similarly, continuous glucose monitoring systems use machine learning to predict fluctuations in blood sugar, given a specific dietary or activity behavior, to provide personalized insights for diabetic patients. Machine learning models advance the interpretation of biologically complex data by filtering noise and pinpointing significant patterns [70,71]. This capability improves the accuracy of biomarker detection in sweat, saliva, or other non-invasive samples, hence making possible the carrying out of more reliable health assessments with fewer false positives and negatives [72]. AI-powered systems can offer personal advice and suggestions based on individual health information and lifestyle [73]. A wearable device, for example, measures the level of physical activity, sleeping, or eating of users to recommend an individualized fitness plan or notify them of possible health risks that may motivate users to engage in proactive health management [74]. AI algorithms excel at finding anomalies in physiological data indicative of health issues or emergent situations [75]. For instance, sudden changes in heart rate or oxygen saturation could be pinpointed with even greater speed to immediately notify the user or healthcare provider. This is where the capability for rapid response can be critical in preventing severe events [76]. Such a machine learning nature therefore means, in real life, models in environments that improve predictive capabilities through continuous learning from newer data [31]. The more users who interact with the biosensors, the more refined the predictions and recommendations of the algorithms, adding more personalization to the system [77,78]. AI will intuitively deliver insights and interactive feedback to enhance wearable biosensors, providing users with optimized interactive engagement [79]. Real-time goal achievement alerts, progress tracking, and tailored health tips will help users stay on track with healthier behaviors through regular self-monitoring [80].

By empowering AI and machine learning, disease diagnosis would be revolutionized with optical and electrochemical sensing technologies in these wearable biosensors. Fluorescence, absorbance, and photoluminescence are some active optical sensors that identify certain biomarkers in body fluids, such as proteins that signal cancers and metabolic indicators of cardiovascular diseases [81]. Electrochemical sensors, for example, convert chemical signals from metabolites or enzymes in sweat, saliva, or interstitial fluid into electrical signals. In this way, continuous monitoring of conditions such as diabetes or metabolic imbalances is possible [82]. When integrated with AI and machine learning, these biosensors will be predictive and analytic, enhancing diagnostic precision, pattern recognition of data over time, and personalization of health insights. AI will keep making such sensors even more sensitive to fine changes in physiology as it learns the health pattern of an individual. The net result is that diagnosis will be earlier, intervention timely, and thus healthcare proactively delivered.

Altogether, AI-integrated wearable biosensors will substantially improve their predictive accuracy and user feedback functionalities. AI-integrated wearable biosensors will substantially improve their predictive accuracy and user feedback functionalities. These technologies enable educated health decisions for individuals and others via data analytics, pattern identification, and personalized suggestions, potentially resulting in enhanced health outcomes and a more proactive approach to personal health management.

### 3.4. Remote Patient Monitoring and the Key Role of Biosensors in Telemedicine and Home Healthcare

The integration of wearable biosensors into telemedicine and home healthcare has transformed the face of patient monitoring into an accessible, efficient, and responsive one. The devices play a very important role in enabling remote patient monitoring (RPM) as a way for healthcare providers to monitor the health metrics of patients outside the conventional bounds of the clinical setting. Wearable biosensors provide an avenue for the continuous monitoring of vital signs, such as heart rate, blood pressure, oxygen saturation, and glucose levels [83]. This real-time monitoring by health professionals will keep them abreast of the patient’s status for timely interventions in cases of detected abnormalities [84]. Considering chronic diseases, such as diabetes, hypertension, and cardiovascular diseases, these wearable biosensors allow individuals to monitor their health continuously. Consistent data collection helps the patient manage their conditions better; moreover, this ensures fewer hospital visits and emergency interventions because abnormalities can be ascertained well in advance by both the patient and healthcare provider for appropriate action [85]. From the aspect of data-driven decision-making, wearable biosensors can collect data from which trends and patterns of a patient’s health are developed over time [86]. This would, in turn, be used in clinical decisions so that further adjustment in treatment plans, according to individual needs and responses, can help in improving health outcomes. By allowing patients to remotely monitor their health, biosensors help to improve involvement in health management [87]. Also, by providing users with direct, instantaneous feedback and insights into their health, such gadgets help to cultivate a treatment-compliant attitude and a better lifestyle. This makes one personally responsible for their well-being. Moreover, this continuous monitoring while at home allows complications to be discovered much earlier, for which the necessary actions can be taken and readmission to the hospital minimized [88]. This is of great use in postoperative patients or even patients with chronic diseases where the complication rate is higher. Biosensors offer access to healthcare for people residing either in the countryside or in faraway places where health facilities are inadequate. These kinds of patients can receive high-quality home-based care; hence, they can manage their health status and quality of life [89]. Wearable biosensors can seamlessly interface with telemedicine platforms, whereby healthcare providers will offer virtual consultations while monitoring patients’ health data in real time [89,90,91]. This again will help bring more effectiveness to the visits to homes and result in more valid assessments, thereby offering personalized care plans. Biosensors are at the forefront of remote patient monitoring, enhancing the effectiveness and accessibility of telemedicine and home healthcare. These devices improve health outcomes and lighten the burden on healthcare systems through continuous health monitoring, chronic disease management support, and fostering patient engagement. Technology will continue to play the catalyst in developing the role of biosensors in RPM, thereby continuously transforming how healthcare is delivered and experienced.

## 4. Applications of Wearable Biosensors

### 4.1. Medical Applications

**Figure 4:**
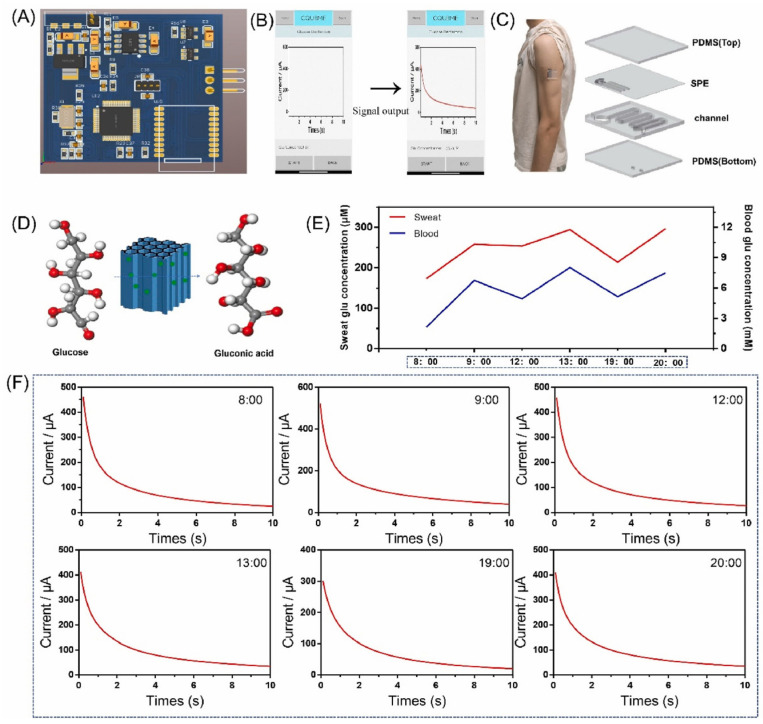
(**A**) Circuit diagram for signal transmission of a POCT device; (**B**) The app installed on a smartphone for receiving and processing electrochemical signals; (**C**) Wearable device affixed to the skin surface; (**D**) Glucose catalyzed into glucuronic acid under the mediation of Co_3_O_4_/rGO/Pt; (**E**) Fluctuations in blood glucose and sweat glucose concentrations before and after meals; (**F**) Detection results of sweat glucose at different time intervals. Copyright Elsevier (2024) [98].

From disease diagnosis to management, wearable biosensors play a vital role in modern medical applications. The capability of continuous real-time monitoring of different physiological parameters helps provide valuable information toward better patient care and clinical decision-making. Wearable biosensors make it possible to identify a number of diseases in their incipient stage by tracking certain biomarkers indicative of specific conditions. Sensors that analyze sweat or saliva, for example, can detect biomarkers for infections, hormonal imbalances, or metabolic disorders, thus enabling timely intervention [92]. ECG sensor wearables can monitor for the presence of arrhythmias or irregular heart patterns. This allows health professionals to identify potential heart problems early through constant cardiac status monitoring, thus improving outcomes [93]. This means, in turn, that the SpO_2_ and respiratory rate sensors can get close to the diagnosis of conditions such as COPD or asthma [94]. Moreover, employing monitoring enables proactive respiratory health management, such as in COVID-19 cases [95]. Continuous glucose monitors (CGMs), due to real-time glucose readings, are very important in the management of diabetes (Figure 4) [96,97,98]. Data will be helpful for both the patient and the healthcare professional in making informed decisions about administering insulin or making dietary choices to minimize the risks of hypoglycemia and hyperglycemia. Wearable biosensors could monitor heart rate, blood pressure, and physical activity and, therefore, be important in managing cardiovascular diseases [99]. Continuous monitoring of these parameters will ensure that patients receive warnings of irregularities for timely intervention and changes in lifestyle that could promote heart health. Smart wearable devices also carry a blood pressure sensor for monitoring hypertension [28]. A device of this nature will give immediate feedback to patients about their levels and will, in turn, automatically prompt them to use their medication and make necessary lifestyle changes. Wearable biosensors can also track physical activity and caloric intake, allowing the individual to maintain a healthy weight and avoid obesity-related diseases [100,101]. These will make perfect devices, tracking activity and adding to dietary monitoring for comprehensive health management. Wearable biosensors remind some patients with chronic illnesses when to take medications on time or warn them upon the non-consumption of a dose [102]. These devices allow better compliance with medication that, in turn, contributes to overall improvement in the management of chronic diseases or health outcomes [103]. Wearable biosensors provide health professionals with the opportunity to monitor vital signs and other measures of health remotely for patients with chronic diseases. This is especially useful for patients who live in remote or inaccessible areas or have mobility problems that often require medical attention [104]. In therapeutic drug monitoring (TDM), wearable biosensors can maintain drugs with narrow therapeutic windows, such as warfarin or insulin, and within that therapeutic range by monitoring fluctuations in drug concentrations and then taking appropriate corrective measures in dosage [105]. Also, wearable technology currently allows doctors to track patients to see if they are sticking to their treatment plans. Transdermal patches and dose consumption detection biosensors are two examples of biosensors that have the potential to improve adherence in patients with chronic diseases such as diabetes and hypertension. Furthermore, these biosensors can be integrated into advanced drug delivery systems, which include closed-loop insulin pumps that measure glucose levels and adjust the amount of insulin released by a pump [106]. Systems such as these are being developed for other drugs too, including painkillers and blood pressure treatments.

Wearable biosensors are among the most revolutionary medical tools being used for diagnostics and therapy in diseases, as well as for the management of chronic diseases. These devices facilitate a shift in health management from passive involvement by the patient to active engagement with the health status of a patient, whereas, for health professionals, there is continuous monitoring with timely interventions. As technology continues to get even better, its applications in health are foreseen to increase, while their benefit in terms of the outcomes and quality of life for patients will significantly improve.

### 4.2. Sports and Fitness and Key Applications in Sports and Fitness

**Figure 5:**
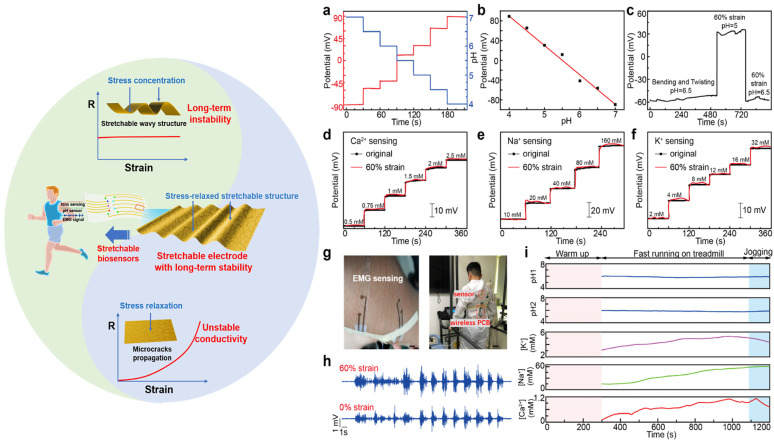
Performance and applications of the multifunctional biosensors. (**a**–**c**) The pH sensor’s performance, including stepped response, linear sensing, and sensing property under deformation and stretch. (**d**–**f**) The open-circuit potential responses to the respective analyte solutions of the Ca^2+^, Na^+^, and K^+^ sensors in their original state and under 60% strain. (**g**) Images of EMG and multifunctional sweat sensor testing. (**h**) Two-channel electromyography signals. (**i**) Multi-signal monitoring of human sweat from warm-up to running. Copyright Elsevier (2024) [109].

Wearable biosensors open up a wide range of great opportunities in the field of sport and fitness for both professional athletes and amateur sportsmen, enabling them to closely follow their performance, recovery process, and injury prevention through real-time collection of data and analysis for better training and overall well-being (Figure 5) [107,108,109]. In terms of physiology monitoring, wearable biosensors monitor the vital parameters of heart rate, breathing rate, and blood oxygen levels during exercise [110]. Based on this kind of analysis, athletes can determine the intensity of training that will keep them within the targeted zones of their heart rate and, hence, achieve their goals more efficiently [111]. Also, devices with integrated accelerometers and gyroscopes further capture movement patterns, distance traveled, speed, and even cadence. This allows the immediate tracking of performance by an athlete in order to change their training programs accordingly [112]. Moreover, advanced wearables can offer biomechanical insights by tracing the angles of joints and motion patterns while running or cycling. It helps improve such techniques and performance in general [113]. Wearable biosensors can measure recovery by monitoring key indicators of heart rate variability (HRV) and quality of sleep [114]. This is applicable to athletes who will then use such information to decide whether they are recovering well enough between workouts and make appropriate decisions regarding the intensiveness of their training [92]. Wearables can give personalized recovery recommendations on rest days or the specific recovery exercises one needs to do to recover based on physiological responses post workout, therefore enhancing overall effectiveness in training and preventing overtraining [115]. Wearables can look at motion mechanics and identify poor forms or movements that are dangerous and most likely to get someone hurt. This is invaluable feedback for athletes trying to fine-tune their form in an attempt to prevent an injury from occurring in the first place [116]. The biosensors, therefore, help the athletes avoid overexertion, one of the most common causes of injury, as they monitor the intensity and volume of the training sessions. It is with these data that the better management of load and adaptation to training regimens are realized [117]. Some of these wearable biosensors include the ability to detect physiological parameters that could indicate injury risk, such as an abnormal heart rate or extensive fatigue, by using real-time alarm functions [118]. Thus, immediate feedback allows athletes to modify their training activities or take advice from experts promptly. In sports and fitness, wearable biosensors have played a critical role in improving performance tracking, recovery monitoring, and injury prevention [119,120]. These help athletes understand in real time and allow for personalized feedback to make informed decisions on how to train smarter, recover effectively, and minimize the risk of injuries. As technology improves, one can expect that wearable biosensors will make an even bigger difference in sports and fitness, which may result in improved athletic performance and overall health.

### 4.3. Wellness and Lifestyle and Key Applications in Wellness and Lifestyle

**Figure 6:**
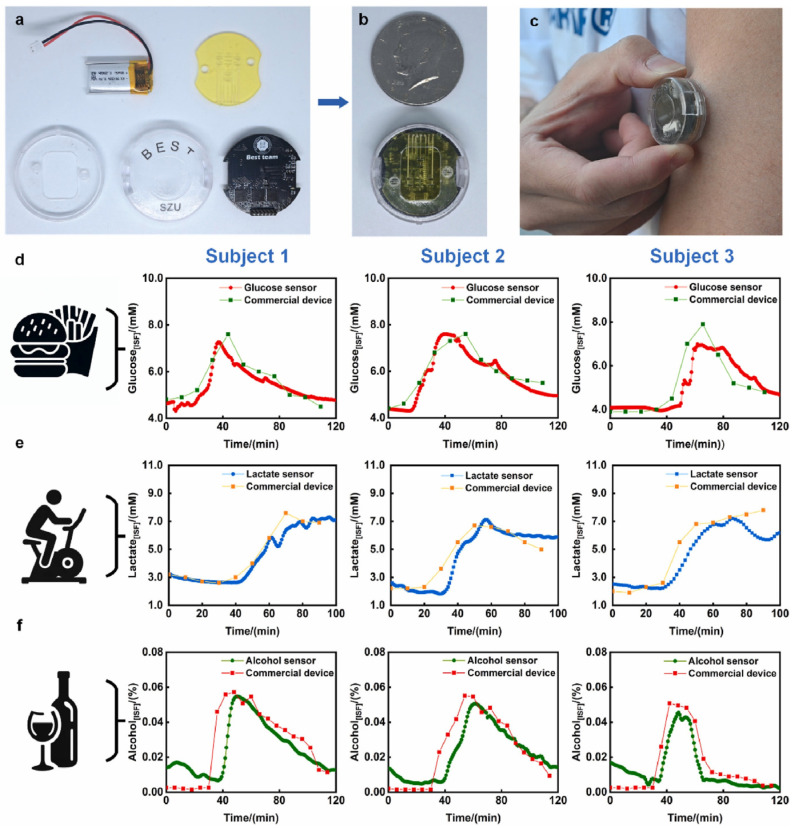
On-body sensing performance. (**a**) Main components of the patch. (**b**) Displaying the assembled patch, comparable in size to a 25-cent coin. (**c**) The integrated microneedle sensor is affixed to the upper arm of the wearer. (**d**) Comparison of ISF glucose concentration (mM) measured by microneedle sensors with reference measurements from commercial glucose blood strips. (**e**) Comparison of ISF lactate concentration (mM) measured by microneedle sensors with reference measurements from commercial lactate blood strips. (**f**) Comparison of ISF alcohol volume fraction measured by microneedle sensors with reference measurements from a commercial breathalyzer. Copyright Elsevier (2024) [110].

Wearable biosensors increasingly monitor many facets of wellness and lifestyle, such as levels of stress, quality of sleep, and hydration (Figure 6) [110]. Providing real-time information on these critical health parameters, these devices enable individuals to make healthy lifestyle choices that will lead to better well-being. Wearable biosensors can measure multiple physiological markers of stress, including heart rate variability (HRV), skin conductance, and cortisol levels [121,122]. Among these, HRV has been found to be a reliable index of autonomic nervous system functioning and is said to reflect response to stress. Most wearables today are able to provide instantaneous feedback to people regarding their stress levels and thereby inform them if their stress levels are increasing [123]. Since it is an instant alert, people can readily avail themselves of the technique to reduce the level of stress through deep breathing, meditation, or physical activity. In that sense, wearable devices can easily let their users monitor their levels of stress over time, recognizing patterns and triggers that will surely enable them to create some effective coping strategies and lifestyle changes to deal with stress [124,125]. In addition, wearable biosensors can monitor a range of sleep parameters that include total sleep time, stages of sleep (light, deep, and REM), and sleep disruptions [126]. Thus, users can study their sleep patterns and pinpoint the conditions that affect quality sleep. Most of these wearables, based on the sleep data, provide suggestions for improvement concerning personal hygiene while proposing ideal sleep schedules, bedtime routines, and techniques for relaxation [127]. Such insight serves to help place better sleep habits in priority order and enhance overall health and productivity [128]. It becomes possible for wearable devices to link sleep data with daily activities, dietary choices, and stress levels; this allows users to understand how their lifestyle is affecting their sleep quality and make informed decisions toward changing it [129]. Some wearables go further and analyze sweat in terms of hydration level and electrolyte balance [130]. The immediate result of these devices would be to give feedback in real-time about fluid needs, especially during exercise or in hot weather. Activity level, environment, and individual physiology could make wearable biosensors recommend personalized hydration goals [131]. By doing this, users will be sure about their optimum level of hydration to maintain physical performance and health in general. For instance, wearables help users develop better hydration habits by tracking hydration patterns. Notifications and reminders about drinking water at regular intervals would promote better hydration throughout the day. Wearable biosensors will also form a very significant part of monitoring stress, sleep, and hydration regarding wellness and lifestyle. This device will make it possible for people to attain real-time data and personalized feedback to enable them to make informed choices that promote better well-being. As technology advances, the integration of these biosensors into daily life will be increased, promoting healthier lifestyles and better quality of life.

### 4.4. Military and Industrial and Key Applications in Military and Industrial Settings

**Figure 7:**
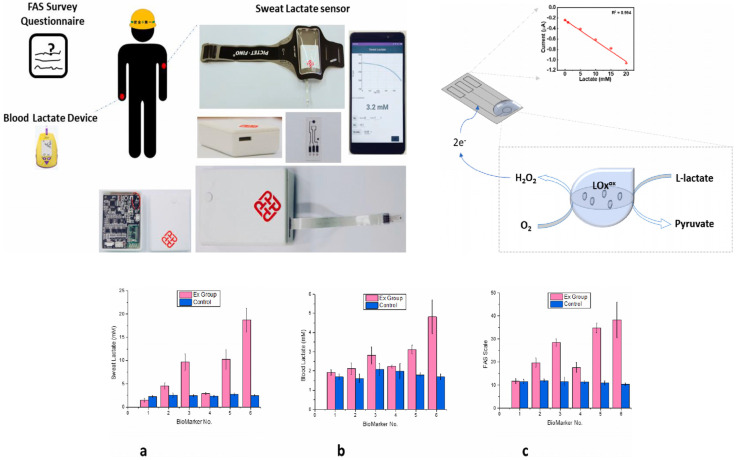
(**a**) Devices for measuring biomarkers using a sweat-based lactate biosensor, a Nova Biomedical blood lactate meter, and a fatigue questionnaire, the fatigue assessment scale (FAS), to obtain biomarkers from participants, including sweat lactate (SL) concentration, blood lactate (BL), and a subjective fatigue score. (**b**) Schematic drawing of the OECT sensor for measuring lactate concentration. (**c**) Comparisons of (**a**) sweat lactate, (**b**) blood lactate, and (**c**) FAS scale between experimental and control groups. Copyright Elsevier (2023) [139].

Wearable biosensors are finding increased application in military and industrial domains, especially in extreme environments where safety and performance monitoring become quite critical. This is itself able to provide physiological and environmental data on a real-time basis, hence increasing operational efficiency and ensuring the safety of personnel at work under demanding conditions. In physiological monitoring, wearable biosensors can offer on-the-spot monitoring of life signs, such as heart rate, body temperature, and hydration levels. Continuous monitoring automatically enables military personnel and industrial workers in very stressful work environments to become more aware of the early onset of their body’s fatigue, heat stress, and other health risk factors [132]. Whereas in environmental monitoring, many wearables provide data about environmental conditions, such as temperature, humidity, and air quality. This information helps personnel understand their surroundings and take precautions to avoid heat-related illnesses or exposure to hazardous substances [133]. Wearable biosensors can provide instant feedback on physiological parameters and allow users to throttle their efforts according to the current state of their physiology. For instance, a military person can know how much endurance and strength to use during training exercises or missions based on instantaneous heart rate and levels of fatigue [134]. Additionally, biosensors could be useful in the development of training programs to maximize performance, such as those used within both military and industry sectors. Physical exertion and its direct correspondence to recovery rates will propel trainer-led programs that are tailored vocationally for the specific operator in a way that only increases operational readiness and efficiency [135]. Wearable biosensors can track motion behavior and identify poor form and/or at-risk behavior that could be signals of injuries [136]. Such feedback is highly relevant to both military training and industrial work tasks in terms of the prevention of musculoskeletal injury and the maintenance of workers’ health in the long run [137]. Workload and physical exertion monitoring will prevent overexertion injuries. Wearables shall monitor the cumulative workload to assist in fatigue management by ensuring the personnel do not exceed their safe physiological limits (Figure 7) [138,139]. Most wearable biosensors can be programmed to alarm when they detect abnormal physiological readings, such as a sudden rise in heartbeats or high fatigue. This allows for real-time intervention, crucial in precluding serious injury or health crisis situations [140]. Wearable devices with communication features will enable better coordination among members of any team in military or industrial operations [141]. Data on health and performance may be shared to enhance situational awareness and collective safety. Wearable biosensors designed for military and industrial use are often ruggedized to withstand extreme environmental conditions concerning temperature, moisture, and physical stress in order to guarantee functionality under the most unfavorable conditions [13,23]. In addition, wearable biosensors are increasingly being applied in the military and industrial sectors to detect hazardous substances, which include nerve agents: potent neurotoxins that pose severe health hazards to humans. The sensors are based on state-of-the-art technologies, including electrochemical and optical detection, able to identify minute traces of a nerve agent present in an environment, alerting an early warning system to avoid exposure to the agents. Wearable biosensors, if they can be developed with sensitive detecting ability, will monitor air quality continuously, detect volatile chemicals, and alert personnel about the presence of dangerous agents in real time [142]. Such devices are the focus of extensive research and development to enhance the safety of high-risk settings for quick responses and to safeguard military and industrial personnel working in environments where toxic exposure risks prevail. It is important for durability in challenging situations. Wearable biosensors and their application in the military and industry have greatly upgraded safety and performance monitoring in extreme settings. These devices provide, therefore, real-time physiological and environmental data to personnel that enable them to make informed decisions, optimize performance, and prevent injuries. In this regard, increased application of wearable biosensors in both sectors will take place in the future with their enhanced capabilities for improved functionality, operational efficiency, and worker safety.

## 5. Future Perspectives

### 5.1. Emerging Technologies in Wearable Biosensors

Some of the budding technologies that are going to play a crucial role in the future of wearable biosensors include hybrid sensors, self-powering biosensors, and implantable devices that will extend both the functionality and application of biosensing technologies, opening up exciting avenues for healthcare, fitness, and wellness. The integration of various sensing technologies will enable hybrid sensors to capture a comprehensive range of physiological signals [143]. Future developments may allow for real-time monitoring of multiple biomarkers, such as glucose, hydration levels, and metabolic rates, simultaneously, providing users with detailed insights into their health status. Such hybrid sensors will also enable personalized health management strategies with advanced algorithms and data analytics [74]. These devices correlate input from various sensors and can make personalized suggestions, thereby rendering health monitoring more relevant and efficient to the individual user [144]. This wearability factor would be completely revamped in self-powered biosensors sans the use of external batteries [29]. Future developments in energy-harvesting technologies such as nanogenerators and biofuel cells will enable these devices to run continuously without stopping, hence allowing proper and continuous health monitoring [145]. Intermittent self-powered biosensors in the context of smart textiles can increase user comfort and compliance [146,147]. Fabrics integrated with energy-gathering wearables offer the possibility of real-time monitoring while becoming part of daily cloth wear, thus embedding health tracking in everyday life [148]. Future devices will also be able to provide more accurate and continuous monitoring of vital signs of health parameters with the increased sophistication of implantable biosensors [22]. For future devices, advanced biocompatible materials and miniaturization might be developed, allowing minimal invasiveness with fewer complications [87]. Fully developed wireless communication technologies have the potential to offer real-time data transmission from implantable devices to healthcare providers for prompt interventions. Real-time data will be especially critical in the management of chronic diseases, as timely data can notify treatment adjustments and emergency responses [149]. The prospect of wearable biosensors, though bright, depends on the complete resolution of many challenges with these emerging technologies. With the development of technologies, regulatory frameworks should change accordingly to guarantee safety and efficacy [103]. Furthermore, guidelines for hybrid sensors, self-powered devices, and implantables have to be laid out in order to win user trust and encourage pervasiveness [150]. Along with increased data collection and sharing, user privacy and security become two of the key issues [151]. Building robust security and transparency of data practices will help reduce apprehensions and gain user confidence in such technologies [152]. With different biosensing technologies still emerging, ensuring that devices and platforms can interoperate will be key to maximizing their potential [153]. In turn, standardization will be required to make data exchange seamless and user experience seamless. Wearable biosensors thus have a bright future, with these emerging technologies pointing toward hybrid sensors, self-powered biosensors, implantable devices, and innovative health monitoring solutions that could revolutionize personal health management to be more proactive from the people’s health management, provided the challenges of the present are resolved and user-centric designs keep the focus. As these devices are researched and developed further, there is no doubt that they will be part of everyday life and thus continue to enable users to live a healthy lifestyle.

### 5.2. Wearable-Implantable Hybrids, Combining Wearables with Implantable Sensors for Comprehensive Monitoring

**Figure 8:**
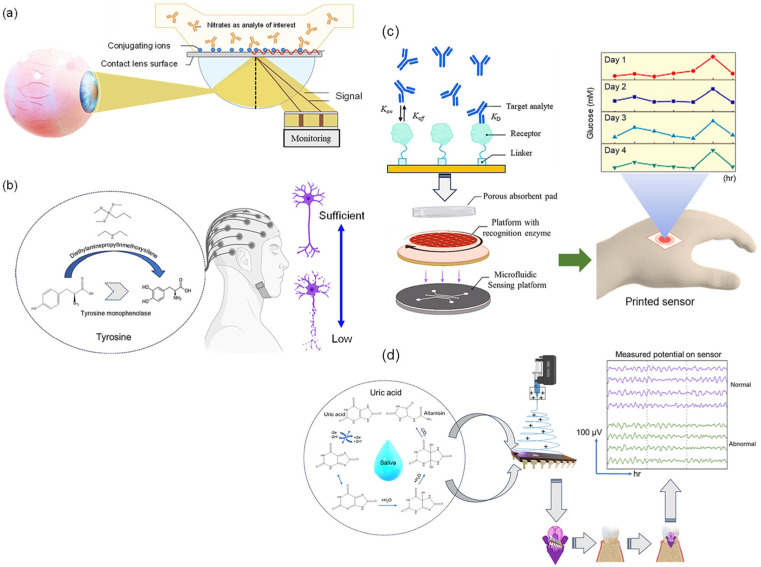
Accessory sensors such as (**a**) contact lenses and eyeglasses and (**b**) headbands have been used for real-time health monitoring, (**c**) an ELISA-based patch-type printed sensor for health sign monitoring, and (**d**) a versatile implantable sensor for real-time health monitoring, drug delivery, and data transmission, with versatile functions. Copyright Elsevier (2024) [154].

The integration of wearable and implantable sensors to track physiological parameters is a big technological advancement in the field of health monitoring, where it looks at inclusivity. Hybrid wearable-implantables, which are popularly known as minimally invasive technologies, could offer an optimal compromise between two of the most influential invasive devices that can break up the way personal health is managed and diseases are tracked. Herein are key views on this emerging technology. These wearable-implantable hybrids can provide continuous monitoring of vital health parameters such as heart rate, glucose level, and oxygen saturation [83]. Sensors implanted within the body, closer to all biological systems, would be able to transmit real-time information, while wearables take external factors and physical activity into consideration, thus building a total picture of a person’s health (Figure 8) [154]. By integrating many sensing technologies, these hybrid devices can obtain the readings of more biomarkers simultaneously [143]. In this way, a device can combine implantable sensor glucose monitoring with a wearable-measured physical activity and stress level, hence showing much more nuanced underlying health dynamics. One of the challenges with wearable biosensors is that the motion artifact can affect the accuracy of the results [155]. Since implantable sensors are anchored internally within the body, they could provide a stable reading unencumbered by external movement, thus potentially amplifying the overall accuracy of health monitoring [156]. Advanced analytics with machine learning algorithms will refine accuracy and predictive capability in health monitoring systems through aggregation from wearable and implantable sensors [157]. These data fusions will help in finding trends and anomalies that may call for medical attention. Comprehensive data coming from hybrid systems enables health providers to provide personalized recommendations on real-time health status that might be able to facilitate proactive intervention, customized treatment, and lifestyle adjustment [158]. Artificial intelligence, embedded within, can enhance decision-making by analyzing huge data from wearable-implantable hybrids. AI algorithms predict health trends and issue personalized alerts so that better decisions may be made on issues to do with the health of the user [67]. Wearables combined with implantables can offer the user the experience of an integrated interface to monitor their health. Users can view notifications and insights on their wearables, while an implantable device will track critical parameters discreetly to promote greater adherence and engagement [141]. It is generally a very empowering process for the individual when they finally have access to comprehensive health data. Easy-to-use apps and interfaces allow users to visualize their health metrics, set goals, and offer words of encouragement that may foster long-term compliance [13]. The hybrid system facilitates remote monitoring by transmitting data in real-time to healthcare providers, thereby enhancing telehealth capabilities and enabling timely interventions and follow-ups for patients with chronic conditions [1]. The incorporation of wearable and implantable technology is anticipated to decrease hospital visits and lower healthcare expenses, thus enhancing healthcare accessibility [159]. While the future for wearable-implantable hybrids seems brilliant, many challenges are yet to be overcome. Wearable and implantable technologies need to be very carefully combined with regard to regulatory approvals [160]. For end-user confidence and wide diffusion, it would be important to demonstrate the safety and effectiveness of hybrid devices. The collection and transmission of sensitive health information will be increasing, thus increasing the importance of data privacy and security [161,162]. Above all, users must have the assurance of security against breaches in their personal information. Development that ensures the complete integration of wearables with implantable sensors and interoperability with existing healthcare will be critical for maximizing the effectiveness of these hybrid devices [163]. The future of wearable-implantable hybrids is huge in comprehensive health monitoring, hence offering an integrated approach to personal health management. These are apt devices that, by the offerings of both wearable and implantable technologies, will provide, on a continuous basis, personalized and precise accurate insights into one’s health status. As technology advances and the challenges are met, wearable-implantable hybrids are sure to transform healthcare by increasing engagement for better outcomes and informed health management.

### 5.3. Next-Generation Applications of Wearable Biosensors

As wearable biosensors continue to evolve, an integration with next-generation applications may well lead to revolutionary healthcare in the form of personalized medicine, prevention of diseases, and even integration with genomics. The future in which monitoring health itself becomes proactive, tailored, and deeply ingrained within the journey of health of the individual. Wearable biosensors can give, in real-time, vital data about health metrics; thus, this enables healthcare providers to tailor appropriate treatment plans in light of the responses of individuals to the therapy [164]. Examples include continuous glucose level monitoring in diabetic patients that may serve as a guide for adjusting insulin dosage and management strategies optimally. In the future, wearable devices may merge genomic information with physiologic monitoring in the delivery of personalized health care [165,166]. Taken together, the genetic information and real-time health information will enable clinicians to construct interventions truly tailored to a patient’s genetic predisposition and metabolic response. Wearable biosensors have great potential for disease prevention because of their ability for continuous collection and analysis of health data. Machine learning algorithms would be able to detect patterns and identify risk factors for early detection of impending health problems [6]. For instance, wearables that can detect abnormal heart rate variability can lead to appropriate preemptive intervention to reduce cardiovascular risks. Wearable biosensors can enable lifestyle changes with feedback on daily activities, sleeping habits, and nutrition [167]. The devices will focus on enabling the user to lead a healthy life based on real-time data to avoid chronic diseases such as obesity, hypertension, and diabetes [168]. In the near future, biosensors integrated into wearable devices will increasingly be informed by genomic information, putting health into a panoramic view [169]. These sensor data can be combined with genetic profiles such that an individual receives valuable insight into how biology influences health and wellness and, therefore, makes more informed choices [170]. Wearable biosensors may be of vital importance in pharmacogenomics, which is a study of how genes affect the response of an individual to drugs [102]. Biomarkers and genetic predispositions, if monitored in real-time, can guide medication selection and dosing to minimize adverse effects and enhance therapeutic efficacy [171,172]. While wearable biosensors are in use, the level of user engagement in health management increases with the level of personalization of insight [13]. With personalized data, there is more potential for being proactive about lifestyle changes, treatment adherence, and enhanced overall health [158]. Social engagement through wearable technology, integrated with social platforms, enhances the sharing of health journeys with others for mutual support and the celebration of milestones, thereby raising motivation and accountability levels in health management [86]. In the future, wearable biosensors will shift the emphasis toward collaborative care models where healthcare providers take an active interest in real-time and improve patient engagement for better outcomes [173]. Remote monitoring will enable timely interventions and continued communication, thus narrowing the gap between patients and providers. Data from wearables can contribute to clinical research and public health endeavors. The aggregated anonymized data will allow researchers to follow trends, comprehend disease progression, and objectively evaluate the efficacy of interventions, thus helping drive medical knowledge [164]. There are a number of challenges that have to be addressed in view of the bright future of wearable biosensors concerning the applications of next-generation biosensors. Wearable data will, through the use of robust management solutions, integrate with genomic and healthcare systems. Ensuring compatibility and seamless data flow between devices, healthcare providers, and genomic databases will be key to fully leveraging these technologies [3,174]. In fact, what that amounts to is some very serious ethical considerations regarding privacy and consent in the gathering and analysis of personal health and genetic data [103]. Data practices will have to be transparent, and there has to be user control over the dissemination of information for general trust in these kinds of wearable biosensor technologies [150]. While wearable biosensors are making their forays into personalized medicine and even genomic data, the regulatory framework needs to upscale similarly for safety, efficacy, and ethical application. This will involve collaboration between the manufacturer, health professional, and regulatory agencies in establishing guidelines [175].

In a broader aspect, screen sensors are the predominant human-machine interfaces utilized in daily life, substantially implemented in personal devices such as mobile phones. Feng et al. [176] recently reported a novel stretchable, on-skin, touchless sensor based on an ionic hydrogel that can achieve gesture detection without direct physical contact. Comfortably attached to the skin, the wearable sensor takes advantage of both the flexibility and conductivity of ionic hydrogels and thus is suitable for wearable electronics and personalized health monitoring. It can even be used for the sensing of hand or object movements through capacitive sensing near the skin for touchless interfaces. The technology here has applications in human-machine interaction, virtual reality, and contactless medical monitoring. This is an innovative approach toward user-friendly interactive wearables.

Personalized medicine, disease prevention, and genomics will be transformational in the next generation of wearable biosensors. Such wearables, relying on real-time data and analytics, will provide one with the ability to manage his or her health proactively, intervene when necessary, and work collaboratively with health professionals. Due to the fact that these technologies will continue to evolve, they will be highly instrumental in changing the future of healthcare and, hence, the health and quality of life of human beings all over the world.

### 5.4. Market Trends and Commercialization of Wearable Biosensors

The wearable biosensor market stands on an edge where transformation will be very rapid, based on radical technological changes combined with increased consumer awareness and new healthcare needs. With the devices starting to find a place in consumer and clinical use, several emerging market trends and commercialization forecasts materialize that will define the future makeup of wearable biosensors. Today, both the consumer and healthcare markets have saturation with continuous glucose monitors and electrochemical sweat sensors for fitness [177]. The integration of AI is also growing in wearables, where some devices already use machine learning to better analyze data and deliver more personalized health insights. On the other hand, self-powered systems that depend on body heat or motion to generate energy are still at the level of research and have not yet entered the commercial mainstream, since the big challenge remains inefficiently sustaining power generation [178]. Other areas of recent development, such as hybrid sensors for multiplexed measurements and implantable biosensor-wearable hybrids, are largely experimental and still under investigation before they can practically be applied.

The wearable biosensor market is set to see robust growth in the coming decade. If any market research reports are anything to go by, the global market should blast past the multibillion-dollar mark come 2030 [179], driven by increasing demand for health monitoring solutions, aging populations, and an increase in chronic diseases. Once the technology matures, applications of wearable biosensors will be found in various sectors such as healthcare, sports and fitness, wellness, and the military [141]. There will be further market expansion as more and more industries realize the worth of continuous health monitoring [88]. Consumer health products will continue to be in higher demand, and within those, wearable biosensors designed to monitor health parameters. Health-conscious consumers looking to optimize their well-being will want devices that measure vital signs, level of activity, quality of sleep, and stress [180]. Wearable biosensors will increasingly be integrated with smartphones, smartwatches, and other connected devices [181]. Stronger integration will lead to improved user experiences by real-time data analysis, providing personalized health insights that will increase consumer involvement and satisfaction. Clinical settings will witness increased acceptance of telehealth and remote patient monitoring, which will increase demand for wearable biosensors [182]. Wearable biosensors will help health professionals monitor the conditions of patients outside the walls of the clinics, improving patient care while reducing the cost of healthcare. As health is shifting to care, wearable biosensors will, in fact, be of utmost importance in managing chronic diseases [159]. Products for the management of diabetes, cardiovascular diseases, and even respiratory diseases will see wider acceptance among both healthcare providers and patients. Continuous development in sensor technologies, materials science, and data analytics will make wearable biosensors even more accurate, reliable, and smaller [174,183]. The latest technological developments will open new perspectives for more miniaturized and, at the same time, resource-efficient devices that can measure a wider range of physiological parameters. Wearable biosensors will further increase their predictive capability with the integration of artificial intelligence and machine learning. Products with the capability of real-time data analysis and delivering actionable insights will be very appealing to both consumers and healthcare professionals [68]. Wearable biosensor commercialization in the future will most likely depend on technology companies, healthcare providers, and research institutions working together [184]. Partnerships will help drive innovation, accelerate product development, and build out ecosystems that enable seamless integration of wearable devices into systems of healthcare. With a growing number of wearable biosensors, data interoperability will increasingly become a foregrounded issue. Companies more attentive to the ease of data exchange between devices and health platforms will bear an advantage in the competitive market [161]. Wearable biosensors look set for a bright future in the market; however, there are a few challenges to successful commercialization. The only way market acceptance will be achieved is by ensuring conformation to the regulatory standards [185]. Manufacturers would need to cope with glibly regulatory mechanisms and become approved for use in health applications. From the perspective of data privacy and security, formidable protection would have to be created against the release of information on users. The responsible sharing of policies about how the data is handled would further instill confidence among both consumers and healthcare professionals [186]. With increasing market size, manufacturers can be assured that competition will get tougher [185,187]. Companies need to create a difference through new features in their products, user experience, and performance if they want to remain relevant in the market [188]. The future of wearable biosensors is characterized by big-market growth and diversification due to rapid technological advancements and changes in consumer preference. With the ongoing development and integration of such devices, their applications in personal health monitoring, preventive health care, and management of chronic diseases will become more pronounced. Based on innovation, collaboration, and security of data, the commercialization of wearable biosensors offers an opportunity for advances in health outcomes and individual self-awareness regarding one’s health. Thus, it will have a great effect in the future of health care as it matures, including new opportunities for better management of health and disease prevention.

### 5.5. Limitations and Challenges

Despite the huge applications of wearable biosensors in many fields, there are a variety of challenges that need to be sorted out from a technical point of view to make them more effective, efficient, and reliable. Some key aspects involve sensor accuracy [174], durability [174,189,190], long-term wearability [158,165], and calibration issues [191,192] having huge effects on performance and user experience. With the increasing integration of wearable biosensors into health monitoring systems, data security and privacy have evolved as the hot focus of concern [193,194,195]. Since such devices handle sensitive health information, strong assurance in security is thus needed to avoid breaches or unauthorized access to such users’ data. Security and privacy in wearable biosensor-based health monitoring systems are of utmost importance to achieve user confidence, which, in turn, enables the profound use of such technologies [159]. With strong security measures in place, with the active promotion of user control over data, and adherence to regulatory standards, stakeholders will be best placed to protect sensitive health data and allow individuals to benefit from wearable biosensors without compromising their privacy. Thus, as technology progresses, every passing day will be filled with vigilance and adaptability regarding the new threats to keep health monitoring systems secure and private. Moreover, special attention is given to several of the most serious regulatory and standardization problems in the course of the development and deployment of wearable biosensors, especially for healthcare. Since these devices are growing in popularity, monitoring health metrics means that ensuring their safety, efficacy, and interoperability is a top priority. Lastly, user compliance is one of the important success factors for wearable biosensors, particularly for applications such as health monitoring. While wearable devices may represent a chance to bring in great benefits, reaching user adoption and long-term maintenance is a task if their full potential is to be tapped. User compliance will be a critical challenge in wearable biosensors for the full realization of their impacts on health monitoring and outcomes [196,197]. Manufacturers and other stakeholders alike should focus on user experience, motivation, relevance of data, issues of privacy, and support systems to establish a long-term engagement where users integrate wearable biosensors into daily life [176,198]. It is under these circumstances alone that wearable biosensors will meet the promise they have for improved health monitoring and overall well-being.

## 6. Conclusions

In recent years, one of the fastest developing areas has been wearable biosensors. Advances in this field have enabled devices to be increasingly sophisticated in their monitoring capabilities. These have included the development and use of many different types of sensing technologies, such as electrochemical, optical, and piezoelectric sensors. Consequent to this, most physiological parameters that indicate health can now be monitored. The application of breakthroughs in material sciences has been further advanced through the use of flexible electronics and nanomaterials, which enable comfortable, long-lasting, miniaturized devices. Artificial intelligence, combined with machine learning, particularly when integrated into wearables, opens up more robust prediction capabilities and bespoke health insights. Applications have expanded to cover a wide range of topics, including illness prevention, real-time health monitoring, and measuring athletic and fitness performance. The promise of wearable biosensors to improve the management of chronic diseases and improve overall health outcomes is becoming more widely acknowledged as these devices continue to evolve.

Wearable biosensors, in the future, might just revolutionize health and even life itself. As technology advances and further assimilates with telehealth and genomics, devices in turn will create a whole new frontier in personalized medicine, using real-time data to develop treatment protocols. The shift toward preventive healthcare will also gain momentum, as wearable biosensors empower individuals to take proactive steps in managing their health and, eventually, improve the quality of life and bring down healthcare costs. Devices will engender health awareness and, therefore, healthier life choices to support a health-consumer society in daily life.

Despite the colossal progress made in wearable biosensors, there is a need to further develop some research activities. First, there is a need for innovative solutions that guarantee data privacy and security. Secondly, further studies are required on user behavior and health outcomes because of continuous monitoring. A few more studies on interoperability and seamless data exchange are also required for the integration of wearable biosensors with healthcare systems. Finally, addressing the regulatory environment will also be necessary for their safe and effective clinical deployment. This will enable future research to continue the advancement of wearable biosensors, realizing their true potential for health and well-being.

## Data Availability

Not applicable.
